# Effectiveness of a psychoeducational intervention on myositis patients’ quality of life and well-being: a randomized controlled trial

**DOI:** 10.1186/s13023-024-03426-0

**Published:** 2024-11-01

**Authors:** Imma Armadans-Tremolosa, Maria Palacin-Lois, Angela Castrechini-Trotta, Susana Sanduvete-Chaves, Salvador Chacón-Moscoso, Albert Selva-O’Callaghan

**Affiliations:** 1 Department of Social Psychology and Quantitative Psychology, PsicoSAO-Research Group in Social, Environmental, and Organizational Psychology, University of Barcelona, Barcelona, Spain; 2 Social Psychology and Quantitative Psychology Department, Faculty of Psychology, University of Barcelona, Barcelona, Spain; 3 Department of Experimental Psychology, Universidad de Sevilla, Seville, Spain; 4 Psychology Department, Universidad Autónoma de Chile, Santiago, Chile; 5 Systemic Autoimmune Diseases Unit, Department of Medicine, Vall d’Hebron General Hospital, Autonomous University of Barcelona, Barcelona, Spain

**Keywords:** HRQoL, Myositis, Inflammatory myopathy, Clinical trial, Psychoeducational intervention

## Abstract

**Background:**

Myositis is a rare disease associated with impaired health-related quality of life. A study evaluating the effectiveness of an intervention to improve the quality of life and well-being of myositis patients is presented.

**Methods:**

All myositis patients in a health district were contacted. Thirty-four eligible patients were randomly assigned to the experimental (n = 17) or control (n = 17) group. A psychoeducational intervention of 5 100-min sessions focusing on the disease as related to daily life was conducted only in experimental patients. Several reliable tools to measure quality of life and well-being were administered twice, before and after the intervention, to both groups.

**Results:**

In the experimental group, post-test scores were higher than pre-test in quality of life, well-being, and self-efficacy to manage the disease. Improvements were more evident in the experimental group than controls in 70% of the variables studied. Specifically, sedentariness decreased and satisfaction with social relationships increased in the post-test evaluation to a greater degree in the experimental group than in controls.

**Conclusions:**

This randomized controlled trial on a representative sample of myositis patients in an extensive population provides evidence indicating the effectiveness of a psychoeducational intervention for improving HRQoL, well-being, and self-efficacy to manage the disease.

*Trial registration*: NCT06300983.

## Introduction

The term *myositis* refers to idiopathic inflammatory myopathies, systemic autoimmune conditions that affect several organs, including lungs, joints, skin, and muscles. These are chronic illnesses with no known cure that cause distress to patients and affect their daily life. There are five recognized phenotypes: dermatomyositis, antisynthetase syndrome, immune-mediated necrotizing myopathy, sporadic inclusion body myositis, and polymyositis. Although specific immunosuppressive therapies may help in managing the disease, they do not provide a cure [1].

As in other chronic diseases, individuals with myositis commonly experience a decline in health-related quality of life (HRQoL) [2–6]. The impact on HRQoL may not be solely attributable to the chronic nature of the disease; it has been suggested that enhancing HRQoL could lead to a more favorable clinical status [7]. In this line, qualitative studies and focus groups have analyzed specific domains, particularly the environmental domain, known to be affected in myositis. Patients have identified certain areas, such as physical activity, a well-established positive modifier of HRQoL, the importance of close relationships with healthcare professionals, lack of acknowledgment of their disease by others and society, and the need for reliable information about the disease, as elements requiring improvement [8].

Thus, the next step is to address these factors to enhance HRQoL and well-being in these patients. Our goal was to assess the effectiveness of an intervention aimed at improving HRQoL in individuals with myositis. The intervention specifically focused on the previously identified areas within the environmental domain of quality of life and involved psychoeducational sessions covering general information about myositis, emotional and occupational issues, personal care, family aspects, affection, and interpersonal relationships.

## Materials and methods

**Fig. 1:**
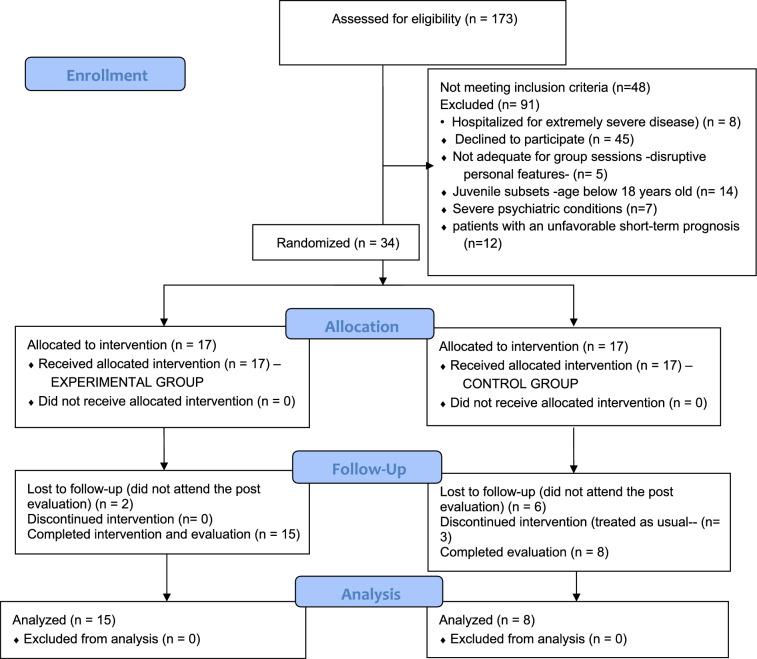
Flow diagram of patient enrolment, allocation, and data analysis

The Medical Research Ethics Committee and Research Projects Commission of the Vall d’Hebron University Hospital approved the project [number PR(AG)398/2022]. Attending to circumstances of the intervention and to enhance methodological quality, the study was designed as a randomized controlled trial (clinicaltrials.gov NCT06300983) with a control group and an experimental group, with two measurement time points [9]. The CONSORT statement [10] was followed to conduct the study (flow chart, Fig. 1).

### Participants

Patients were eligible for enrollment based on the following inclusion criteria: (1) a definite diagnosis of myositis according to the International Myositis Classification Criteria (probability ≥ 90%) [11] and (2) the ability to understand the purpose and procedures of the study, and motivation and agreement to participate.

A cohort of 173 adult myositis patients attending our outpatient clinic (Systemic Autoimmune Diseases Unit of Vall d’Hebron General Hospital, Barcelona, Spain) were potential candidates for the study. Vall d’Hebron General Hospital is a 700-bed referral and teaching hospital for a catchment population of nearly 450,000 inhabitants. Virtually all patients from the area with suspected myositis are referred to Vall d’Hebron, where they are diagnosed, treated, and followed up, whether the disease is severe or not.

Disease activity, routinely evaluated at all medical visits, used a 10-cm visual analog scale (VAS) anchored with the descriptors *no activity* (0) and *maximum activity* (10), the Myositis Disease Activity Assessment Tool (MDAAT) which records the physician’s assessment of disease activity in various organ systems (Global Disease Activity, with a VAS range from 0, *no activity*, to 10, *maximum activity*), and the manual muscle testing procedure (MMT8) to determine muscle weakness, performed unilaterally in eight muscle groups (axial, proximal, and distal), which runs from 0, *maximum weakness*, to 80, *normal strength* [12]. Scores were retrospectively retrieved from the patients’ charts.

All candidate patients were contacted by telephone and invited to participate in the study during a 2-month period (September and November, 2022). In total, 139 patients were excluded from the study. Among these, 48 did not meet the diagnostic criteria and an additional 91 were excluded due to the following reasons: hospitalized for an extremely severe illness, declined to participate, required excessive convincing for participation, deemed likely to play a disruptive role in the group, younger than 18 years, had severe psychiatric conditions, or had an unfavorable short-term prognosis.

Thirty-four eligible patients according to the study criteria were randomly allocated to the experimental and control groups using an online resource (https://recursostic.net/generador-de-grupos-aleatorios/): 17 patients were assigned to the experimental group (40% women, mean age 51.13 years, 73.3% diagnosed with dermatomyositis), and 17 patients to the control group (62.5% women, mean age 55.13 years, 62.5% with dermatomyositis).

### Psychoeducational intervention

The psychoeducational program was carried out in the experimental group (17 myositis patients) during September and October, 2022. The other 17 patients (controls) did not participate in the intervention, were treated as usual, and were placed on a waiting list to receive the psychoeducational intervention after completion of the study. Patients who received the intervention had no contact with the control participants (Fig. 1). The intervention consisted of 5 sessions (Table 1), with each lasting 100 min and delivered on a weekly basis. Individual meetings were conducted with each participant prior to starting the group work. These were considered pre-intervention sessions, during which patients provided informed consent to participate in the study and the first assessment tests were administered.

**Table 1:** Summary of the five psychoeducational intervention sessions

Initiation stage		Development stage		Closure stage
Communication level interpersonal and cognitive		Communication level interpersonal-ingroup and emotional		Communication level ingroup and cognitive-emotional
Session 1	Session 2	Session 3	Session 4	Session 5 (post psychometric tests phase)
*Psychoeducational program thematic content*				
1. Presentation Program 2. Disease and Symptomatology 3. Necessary Habits and Care	1. Physical and Leisure Activities 2. Work Activity and Emotion	1. Family and Social Support 2. Affectionate Relationships	1. Work and Society 2. Social Significance and Proactivity	1. Evaluation and Assessment
*Group process in each session*				
Trust	Trust	Conflict-cohesion	Cohesion-cooperation	Cooperation
*Duration of each session*				
100 min	100 min	100 min	100 min	100 min

Two specialized professionals participated in the group sessions: a leader and an external observer present during the process [13]. The leader positioned herself within the group ecology, facilitating the dynamics and development of the sessions. The observer, located outside the group ecology, but in the same room, was responsible for preparing complete minutes of each session and detailed observations, recording the topics discussed and the session structure, as well as presences, absences, delays, and critical incidents, among other relevant data [14].

The duration and structure of each session was constant, with an introduction phase of 30 min, a central phase (the most substantial part of each session) of 50 min, and a final phase of 20 min. The methodological approach used in the study allowed for systematic and consistent implementation of the group psychoeducational program. The combination of individual and group sessions, together with the presence of an external observer, provided a comprehensive view of the progression of the participants and the topics covered. The content of the intervention is summarized in Table 2.

**Table 2:** Psychoeducational intervention

*Session 1. Presentation, illness, habits and care*
a. Presentation of the professionals’ technical roles: leading the intervention and observation
a. Presentation of *idiopathic inflammatory myopathy* (what is this disease, symptoms, progression, recommended habits and care)
b. Presentation of the participants (10 in total; structured format, 2 min per participant)
c. Use of a technique to identify healthy and unhealthy habits related with the disease present in the patients’ lives (structured format, 2 min per participant)
d. Identification of recommended care in the patients’ lives, using a participatory approach to ensure that the information is correct and clear
e. Patients commit to an individualized care plan they will carry out for the next session
f. Session closing: Patients reflect on their feelings using a single word
*Session 2. Work activity and emotions*
a. Introduction. The timeline of the disease course and instructions to perform the technique: incidents during the course of your disease
b. Explanation by each participant of the disease timeline: they are asked to discuss changes in various areas of their life, including work, physical aspects, and leisure
c. Participants identify and select two behaviors to perform in each area listed above for their own benefit
d. Task for the next session: bring photographs of the most important people in 3 levels (work, leisure, and friendship)
e. Session closing
*Session 3. Family and affective relationships*
a. Introduction: The family and its importance in adapting to the disease
b. Discussion of the types of social support (instrumental support, affective or emotional support, and material support) and the types of emotional relationships (are they reciprocal/non-reciprocal)
c. Explanation of the photograph selected (task proposed in the previous session) relating it to the types of support and types of affectionate relationships (structured format, 5 min per participant)
d. Session closing
*Session 4. Work and society*
a. Introduction: The role of work activity and the feeling of social usefulness and adaptation to the disease
b. Discussion of the social significance in patients’ lives following the disease diagnosis
c. Explanation of intragroup relationships and the importance of support networks in rare diseases
d. Participants’ explanations regarding these networks (structured format, 2 min per participant)
e Discussion on social understanding and the influence of social norms in relation to various areas
f. Session closing
*Session 5. Evaluation and assessment*
a. Introduction of the session
b. Evaluation and assessment by the participants
c. Psychometric tests
d. Individual assessment of the psychoeducational group intervention (structured format, 5 min per participant)
e. Group Farewell and Closing

### Measures

Participants were required to fill out a series of questionnaires related with the objectives of the study at 2 time points: before the start of the intervention and on the last day of the intervention. The control group, which did not participate in the intervention, filled out the same questionnaires at the same 2 time points [15, 16]. The battery of questionnaires included the following scales:

*World Health Organization Quality of Life Measure (WHOQOL-BREF*): a comprehensive, generic questionnaire designed to evaluate various aspects of quality of life. The WHOQOL-BREF consists of 26 items, with 24 items covering four domains: physical health, psychological health, social relationships, and environment. Additionally, 2 global questions inquire about overall quality of life and satisfaction with health. Participants rated each item on a 5-point scale, in which higher scores indicate better quality of life. The assessment was based on the experiences of the past 2 weeks. Results from the 4 domains multiplied by 4 gives a score from 0 to 100, with higher scores indicating better quality of life.

The Spanish version of the WHOQOL-BREF has demonstrated good psychometric properties among elderly Spanish individuals, validating its suitability for the study population [17, 18].

*World Health Organization Well-Being Index (WHO-5)*: a five-item scale used to assess positive well-being experienced over the past 2 weeks. Each item is rated on a 6-point Likert scale, ranging from 0 (indicating *at no time*) to 5 (representing *all of the time*). The total score ranges from 0 to 25. The total multiplied by 4 gives a score from 0 to 100, with higher scores indicating a greater sense of well-being. The WHO-5 has shown high reliability [19].

*Self-Efficacy to Manage Chronic Disease Scale (Spanish version, SEMCD-S):* developed and validated to assess self-management following an intervention conducted in Stanford University's Chronic Disease Self-Management Program. The questionnaire comprises 4 items, and participants rate each item on a scale from 1 (*very unsure*) to 10 (*very sure*). The self-efficacy value is determined as the average of the 4 scores obtained. Higher scores on the scale indicates higher levels of self-efficacy. The questionnaire has shown favorable psychometric properties, indicating good reliability [20].

*International Physical Activity Questionnaire—Short Form (IPAQ)* (validated Spanish short-form version). This questionnaire assesses types of physical activity according to their intensity levels and the amount of time spent sitting, as individuals engage in their daily routines. These factors are used to estimate the total physical activity in MET-minutes per week and the duration of sitting time. The tool includes inquiries about 3 types of activity—walking, moderate-intensity activities, and vigorous-intensity activities—performed over the last 7 days, along with a question about sitting time. The overall physical activity (PA) score is obtained by summing up the MET-minutes per week for all 3 types of activities. The study classifies the population into 3 PA levels—low, moderate, and high—following the IPAQ guidelines [21]. Test–retest reliability has indicated good stability [22].

Participant information encompassed sociodemographic data, which comprised age, sex, marital status, household composition, educational background, and employment status. Clinical data, such as age at diagnosis, specific diagnosis, and years of disease follow-up, were also recorded.

### Statistical analysis

SPSS version 28 (SPSS Software Inc., Chicago, IL) was used for the statistical analyses. The McNemar test was carried out for nominal dependent variables, and repeated measures ANOVA was used for quantitative variables [23]. Eta squared was used as a measure of effect size. Effect size values around 0.01 were considered a small effect, around 0.06 a medium effect, and around 0.14 a large effect.

## Results

### Participant characteristics

Characteristics of the study participants are reported in Tables 3 and 4. Patients in the experimental and control groups did not differ with regard to the pre-test sociodemographic characteristics or clinical data. Most participants in the experimental group successfully completed the intervention (N = 15, 88.2%). There were no differences in pre-test sociodemographic or clinical variables between those who completed the intervention and those who dropped out. In the control group, 8 participants (72.7%) completed both measurements.

**Table 3:** Characteristics of study participants

Sex, female	Experimental (N = 15)		Control (N = 8)	
	6	40%	5	62.5%
Age, mean (SD)	51.13 (7.55)		55.13 (10.45)	
Civil state				
Single	3	20%		
Married	9	60%	7	87.5%
Divorced	3	20%	1	12.5%
Family unit				
Family (couple and any child)	8	53.3%	8	100%
Mother	1	6.7%		
Couple	4	26.7%		
Children	1	6.7%		
Alone	1	6.7%		
Education				
University	4	26.7%	6	75%
Technical college degree	1	6.7%		
Secondary	6	40%	2	25%
Primary	4	26.7%		
Work status				
Active	6	40%	4	50%
Retired	6	40%	4	50%
Unemployed	3	20%	0	0%
Diagnosis				
Dermatomyositis	11	73.3%	6	75%
Antisynthetase syndrome	3		1	12.5%
Immune-mediated necrotizing	1	20%6.7%	1	12.5%
Myopathy				
Age at diagnosis, mean (SD)	37.53 (17.52)		48.13 (14.36)	
Follow-up years, mean (SD)	13.07 (12.64)		6.25 (3.54)	

Data are reported as n (%) unless otherwise specified

**Table 4:** Clinical, immunological and pathological characteristics of participating myositis patients experimental group (n = 15)

Phenotype	Activity (VAS/MDAAT/MMT)*	Ab profile	Skin disease	IS	Muscle pathology	Disease duration, y	Highest CPK peak^#^
1. CADM	1/1/80	MDA5 (+)	(++)	GC	N/A	5	113 IU/L
2. ASS	3/3/80	Jo (+) Ro (+)	(+)	GC/MFM/RTX	N/A	4	2755 IU/L
3. ASS	0/0/80	Jo1 (+)	(-)	GC/MFM	N/A	5	801 IU/L
4. DM-CA	0/0/80	TIF1γ (+)	( ±)	Topical GC	DM	11	1134 IU/L
5. DM	2/2/75	TIF1γ (+)	(+)	GC/MFM	DM	1	304 IU/L
6. DM	3/3/80	Jo (+) Ro (+)	(++)	GC/MFM/LFN	DM	34	150 IU/L
7. DM	0/0/80	NXP2 (+)	(-)	(-)	DM	20	1905 IU/L
8. DM	0/0/80	SAE (+)	(±)	GC/MFM/IVIG	DM	9	359 IU/L
9. IMNM	3/3/70	SRP (+)	(-)	GC/IGIV	IMNM	11	1600 IU/L
10. CADM	1/1/80	MDA5 (+)	(+)	GC/MFM	N/A	7	105 IU/L
11. DM	1/1/80	PM/Scl (+)	(+)	(−)	DM	20	93 IU/L
12. DM	0/0/80	PM/Scl (+)	(−)	MFM	DM	39	456 IU/L
13. DM	1/1/75	PM/Scl (+)	(+)	GC	DM	5	1217 IU/L
14. DM	0/0/80	SAE (+)	(±)	GC/MFM	DM	4	> 1000 IU/L
15. ASS	0/0/80	EJ (+) Ro (+)	(−)	GC/TAC/RTX/IVIG	ASS	3	2890 IU/L

Control group (n = 8)							
1. DM	0/0/80	(−)	(−)	GC MFM	DM	9	1638 IU/L
2. DM	0/0/80	PM/Scl (+)	(−)	GC MFM	DM	5	1365 IU/L
3. IMNM	0/0/75	(−)	(−)	GC MFM	IMNM	3	3737 IU/L
4. DM	2/2/80	TIF1γ (+)	(+)	GC/IVIG/TAC/ LFN	DM	4	9913 IU/L
5. DM	0/0/75	(−)	(−)	GC/MFM	DM	16	19,908 IU/L
6. DM	0/0/80	(−)	(−)	GC	DM	12	356 IU/L
7. DM	2/2/80	(−)	(+)	GC/CyA	DM	7	4500 IU/L
8. ASS	1/1/75	PL7 (+)	(+)	GC/MFM	ASS	2	189 IU/L

*Activity recorded at the beginning and end of the study, with non-significant differences between the two periods

^#^Normal value, 45-195 IU/L. ASS, antisynthetase syndrome; CADM, clinical amyopathic dermatomyositis; CPK, creatine phosphokinase; CyA, cyclosporine; DM, dermatomyositis; DM-CA, cancer-associated dermatomyositis (breast cancer in patient 4, experimental group, bladder cancer in patient 4, control group); GC, low-dose glucocorticoids (≤ 7.5 mg/d); IMNM, immune-mediated necrotizing myopathy; IS, immunosuppressive drugs; IVIG, intravenous immunoglobulins; LFN, leflunomide; MFM, mycophenolate mofetil; SSc, Systemic sclerosis; RTX, rituximab; TAC, tacrolimus; VAS/MDAAT/MMT, Visual Analogue Scale/Myositis Disease Activity Assessment Tool/ Manual Muscle Testing

### Intervention effectiveness

Means and standard deviations for outcome measures in the pre- and post-tests for the experimental and the control groups are shown in Table 5. In the experimental group, all variables measured obtained an improvement in post-test when compared with pre-test. Although a slight improvement was also seen in most variables in the control group, the improvement obtained in the experimental group was higher in 70% of the variables studied. In total, the post-test score was better in the experimental group than the control group in 80% of the variables studied.

**Table 5:** Scores for outcome measures in the experimental and control group (pre-test and post-test)

Outcome variables	Experimental group (N = 15) Mean (SD)		Control group (N = 8) Mean (SD)		*p*
	Pre-test	Post-test	Pre-test	Post-test	
Physical activity^a^ (IPAQ)*	2763.46 (1999.58)	3805.57 (3110.39)	3325.41 (5428.71)	4325.36 (5203.14)	.384
Sedentariness^b^ (IPAQ)*	7 (3.19)	6.6 (3.23)	5 (3.30)	5.5 (3.38)	.341
Quality of life* (WHOQOL—item1) [1–5]	3.53 (1.06)	3.73 (0.96)	3.13 (0.84)	3.25 (0.71)	.882
Satisfaction with health* (WHOQOL—item2) [1–5]	3.13 (0.915)	3.53 (0.743)	2.38 (0.518)	2.62 (0.744)	.642
Physical health (WHOQOL—Domain 1) [0–100]	65.07 (16.312)	66.40 (20.900)	49.13 (11.154)	50.75 (13.079)	.931
Psychological health* (WHOQOL—Domain 2) [0–100]	64.67 (18.965)	67.13 (22.564)	53.88 (8.079)	55.50 (9.710)	.850
Social relationships* (WHOQOL—Domain 3) [0–100]	56.20 (22.431)	60.40 (17.776)	48.50 (5.318)	48.38 (10.336)	.477
Environment* (WHOQOL—Domain 4) [0–100]	70.07 (15.327)	73.93 (15.494)	62.00 (11.301)	63.50 (9.710)	.580
Wellbeing (WHO-5) [0–100]	57.07 (25.047)	61.33 (18.247)	51.50 (13.256)	58.00 (10.690)	.701
Self-efficacy (SEMCD-S) [1–10]	6.67 (2.167)	6.78 (1.932)	5.06 (1.976)	5.69 (1.792)	.379

^a^Physical Activity (IAPQ) is measure in MET: minutes per week

^b^Sedentariness (IPAQ) is measured as the duration of sitting time: hours per day

*The improvement obtained in the experimental group was higher than in the control group

With regard to sedentariness, there was an improvement in the experimental group and a slight worsening in the control group, with a small/medium effect size, although the difference between groups was not statistically significant: *F* (1, 21) = 0.948, *p* = 0.341, *η*^2^ = 0.043. This tendency also occurred in reference to social relationships, where there was an increase in the experimental group and a slight decrease in the control group, with a small/medium effect size, but showing nonsignificant differences: *F* (1, 21) = 0.524, *p* = 0.477, *η*^*2*^ = 0.024. Finally, a small/medium effect size was also found for self-efficacy, where there was an improvement in the post-test results—*F* (1, 21) = 0.809, *p* = 0.379, *η*^2^ = 0.037—although a statistically significant difference between groups was not attained.

The two variables analyzed with the McNemar test (physical activity—low, medium, high; and sedentariness—yes, no) did not present relevant differences between the experimental and control group: *Χ*^*2*^(3) = 3.8, *p* = 0.384 and *Χ*^2^(3) = 0, *p* = 1.000, respectively.

## Discussion

In this controlled trial with randomization, we assessed the impact of a psychoeducational intervention on HRQoL and well-being in patients with myositis. We observed a modest enhancement of HRQoL and well-being in both the experimental and control groups. However, there was a somewhat greater, although statistically non-significant improvement in the experimental group, where the intervention was implemented, compared to the controls. Our findings are in line with previous research reporting a significant enhancement in quality of life through the use of educational interventions [24]. In addition, it is well-recognized that studies and educational interventions constitute an adequate resource to achieve lifestyle changes. To the best of our knowledge this study is the first to evaluate a psychoeducational intervention specifically targeting patients with myositis.

Several factors should be considered when interpreting the findings. The limited sample size, due in part to the low frequency of the disease and to the strict inclusion criteria for entering the trial, allowed us to analyze only a small sample of patients and controls. This is a shortcoming of the study. Nonetheless given the rarity of the disease, with an incidence ranging from 0.2 to 2 cases per 100,000 inhabitants/year worldwide [25], our results can be of value.

It should be noted that while some of the variables examined showed improvement in the control group, likely due to a placebo effect, the experimental group generally exhibited better post-test scores for most variables. The dependent variables assessed are characterized by showing a gradual change that manifests progressively over time. The absence of worsening and detection of a change, although not radical, indicate the suitability of the intervention, especially considering the relatively short time interval between measurements. Data from other authors support an enhancement in quality of life and well-being following a group-based intervention, reducing negative affect and promoting mental health and optimism, particularly in the long term [26].

The intervention also led to positive changes in physical activity and sedentariness, two measures of paramount relevance in myositis patients. The improvements in these variables are important, as existing knowledge indicates that physical activity contributes to a better prognosis in myositis patients [27]. Thus, interventions focused on the environmental domain, aiming to enhance physical activity and reduce sedentary behavior, could generate a virtuous circle and lead to better prospects for these patients.

The success of this psychoeducational intervention lies in its targeted approach to specific domains previously identified through qualitative studies and focus groups [8]. By addressing emotional, occupational, personal care, family, and affection-related issues, the program aimed to comprehensively tackle the HRQoL environmental domain affected in myositis. Our findings suggest that this tailored approach can significantly enhance patients' life experiences, contributing to a more positive outlook. Notably, the intervention had a substantial positive impact on social relationships, with the experimental group exhibiting increased satisfaction in this area. These outcomes are particularly promising, as social isolation has been identified as a significant challenge for myositis patients. A positive example of the impact of the intervention on this factor is the establishment in our setting of an ongoing association for individuals with myositis, whose core members are patients that participated in the psychoeducative program. Furthermore, our results may contribute to improving the effects of the disease [7]. Improvements in aspects related to quality of life and well-being have been documented in other conditions, including a reduction in sedentary lifestyles, satisfaction with social relationships [28], and self-efficacy for managing the disease.

## Study limitations

While the positive results obtained here are encouraging, the low incidence of the illness in the population has resulted in a small sample size, reducing the statistical power. During the study design, we debated whether to additionally include patients with a *probable* IIM diagnosis according to the ACR/EULAR criteria. However, we decided against it to maintain a more homogeneous sample. This decision does not imply that the intervention would be effective only in patients with definite IIM. It is very likely that individuals with probable IIM would also benefit, and we consider this a worthwhile area to explore in future research. Furthermore, although it is widely acknowledged that disease activity can impact well-being and HRQoL [2, 5], the fact that the majority of the sample consisted of stable patients attending our myositis outpatient clinic precludes drawing firm conclusions in this regard. Additionally, the short-term nature of the intervention and follow-up period require future comparisons of the findings with the long-term effects observed in different patient cohorts. Finally, the use of qualitative assessments with mixed methods to study the participants' subjective experiences could provide deeper insight into the mechanisms through which psychoeducation influences HRQoL and well-being.

## Conclusions

In conclusion, the findings of this study offer evidence supporting the effectiveness of a psychoeducational intervention to enhance HRQoL, well-being, and self-efficacy in myositis patients. The study provides an extensive description of the patients and setting, details the sampling techniques used in the target population, and specifies the components of the intervention program. Summing up, the intervention had a positive impact on the patients’ personal and social skills, social relationships, and sedentary behavior. These findings highlight the potential of targeted psychoeducation as a valuable component in the management of chronic conditions such as myositis. Further research is needed to confirm and expand upon these findings, ultimately contributing to the development of comprehensive, patient-centered interventions for individuals living with myositis.

## Data Availability

The datasets used and/or analyzed during the current study are available at https://osf.io/cj6qx/?view_only=9fe53d45728043c49bdad72f7aa68c40
